# Amide‐Linked Local Anesthetics Alter Tumor Biology in a Complex Human Tissue Model of Non‐Small Cell Lung Adenocarcinoma

**DOI:** 10.1002/adbi.202500280

**Published:** 2025-09-30

**Authors:** Juliane Krömer, Sebastian Krämer, Ngoc Anh Hoang, Doreen Sittig, Isabella Metelmann, Uta‐Carolin Pietsch, Sebastian N. Stehr, Sonja Kallendrusch, Tobias Piegeler

**Affiliations:** ^1^ Institute of Anatomy University of Leipzig Liebigstraße 13 04103 Leipzig Germany; ^2^ Department of Anesthesiology and Intensive Care University of Leipzig Medical Center Liebigstraße 20 04103 Leipzig Germany; ^3^ Department of Visceral, Transplantation, Thoracic and Vascular Surgery University of Leipzig Medical Center Liebigstraße 20 04103 Leipzig Germany; ^4^ Institute of Clinical Research and System Medicine Health and Medical University Potsdam Schiffbauergasse 14 14467 Potsdam Germany

**Keywords:** cancer, local anesthetics, perioperative oncology, tumor biology, tumor microenvironment

## Abstract

Amide local anesthetics (LA) affect tumor burden in various preclinical studies, possibly via their anti‐inflammatory properties. However, a translation into clinical evidence is still lacking. Here, effects of LA at clinically relevant concentrations are assessed using a human ex vivo tumor model of patient‐derived tumor slice cultures from nine patients with non‐small cell lung cancer. Tumors are cultivated for four days and treated with LA in absence/presence of cisplatin. Tumor cell proliferation and apoptosis as well as expression of intercellular adhesion molecule‐1 and macrophage polarization are assessed using immunofluorescent imaging. Tumor specimens are considered to be “Responders”, when a change in proliferation and/or apoptosis by >50% compared to untreated slices occurred. Five of nine samples are “Responders”, in which the LA exerted effects comparable to cisplatin. Even at clinically relevant concentrations of LA, a strong anti‐tumoral effect is observable in patient‐derived tumor slice cultures with complex structures of the tumor microenvironment highlighting the LA effect on the tumor itself and its surroundings, without any interference by other leukocytes or neuronal stimulation. The diverse reaction to LA treatment also emphasizes the importance of biomarkers to determine the tumor phenotypes, which may benefit from LA treatment.

## Graphical abstract

Amide local anesthetics (LA) affect tumor burden in various preclinical studies, possibly via their anti‐inflammatory properties. However, a translation into clinical evidence is still lacking. The current study demonstrates that LA ‐ even at clinically relevant concentrations – exert a strong anti‐tumoral effect in a complex ex vivo model patient‐derived tumor slice cultures with complex structures of the tumor microenvironment and its surroundings.

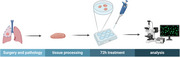

## 1 Introduction

Local anesthetics (LA) have gained increasing attention in the field of “perioperative oncology” in recent years. Beyond their established mechanism of action involving the blockade of voltage‐gated sodium channels,^[^ ^1^ ^]^ LA have demonstrated the ability to modulate a wide field of ion channels, G‐protein coupled receptors as well as inflammatory signaling pathways across various cell types.^[^ ^2^, ^3^, ^4^, ^5^, ^6^ ^]^ Although the specific mechanisms of action of LA on these pathways are not yet fully understood, a growing number of studies indicate that the type of anesthesia may exert a significant influence on patient outcomes following tumor surgery.^[^ ^7^, ^8^ ^]^ Yet, surgical removal of the tumor still represents a cornerstone of various cancer treatments, especially in lung cancer. However, during complete tumor resection, circulating tumor cells might persist and initiate recurrence or metastasis, while the setting of biopsy sampling or a debulking with incomplete resection are completely different in this regard. In the latter, the complex tumor microenvironment (TME) persists and is still able to interact with the tumor, thus continuously promoting growth and metastasis.^[^ ^9^ ^]^ However, the TME might also be an interesting target for anti‐cancer interventions, such as the application of amide LA: The drugs might exert specific effects on stromal cell populations, e.g., tumor associated‐macrophages (TAM), as these cells carry certain ion channels and GPCR receptors, which might be relevant targets for LA.^[^ ^10^, ^11^, ^12^ ^]^

Recently, we were able to show that LA inhibit tumor necrosis factor (TNF)‐α mediated pro‐inflammatory signaling pathways in human lung adenocarcinoma cells,^[^ ^13^ ^]^ thus preventing the activation of Src tyrosine protein kinase (Src).^[^ ^14^ ^]^ Src activation not only impairs endothelial integrity by interacting with intercellular adhesion molecule‐1 (ICAM‐1) and by disrupting tight junctions, but also promotes migration and invasion in cancer cells.^[^ ^15^ ^]^ Macrophages as part of the TME also play an important role in these inflammatory signaling events and might therefore also be affected by the LA.^[^ ^16^, ^17^ ^]^ A certain need to monitor local response patterns to determine the effectiveness of treatments of tumors with LA in the heterogeneous TME might therefore be warranted and of utmost interest in order to determine the LA mode of action.

However, the translation of previous findings – especially from 1D in vitro or in vivo models – to the complexity of tumor biology is not readily feasible.^[^ ^18^, ^19^ ^]^

In order to address the direct effect of LA on malignant cells within a complex TME in a translational, exploratory approach, we investigated the potency of LA in patient‐derived tissue cultures (PDTCs) of human non‐small cell lung adenocarcinoma, without the confounding neural activity of an in vivo setting.^[^ ^16^, ^20^, ^21^ ^]^ Anti‐tumor effects of amide LA lidocaine and ropivacaine as well as a potential synergistic effect with the chemotherapeutic agent cisplatin were assessed. In order to generate hypotheses for future research projects, the expression of ICAM‐1 and the polarization of tissue resident macrophage populations were also investigated in this exploratory study as both structures might directly be targeted by LA.^[^ ^16^ ^]^

## 2 Experimental Section

Tumor specimens were obtained from patients treated at the University of Leipzig Medical Center (Leipzig, Germany). Specimens from a total of 12 patients with NSCL adenocarcinoma (NSCLaC) were assessed for this study. PDTCs derived from three patients had to be excluded from the final analysis as no tumor or only large necrotic areas were found at baseline, leaving a total of nine PDTCs for final analysis. No patient received neoadjuvant treatment prior to surgery. The scarcity of available tissue prevented testing all conditions for each PDTC. Limited tissue resources required selective testing and limited the experimental scope. The study was approved by the Ethics Committee of the Medical Faculty, University of Leipzig (Protocol No. 370–1316122013). Written informed consent was obtained from all patients involved in this study. All research was performed in accordance with the Declaration of Helsinki.

### 2.1 Preparation of Patient‐Derived Tissue Cultures (PDTC)

**Figure 1:**
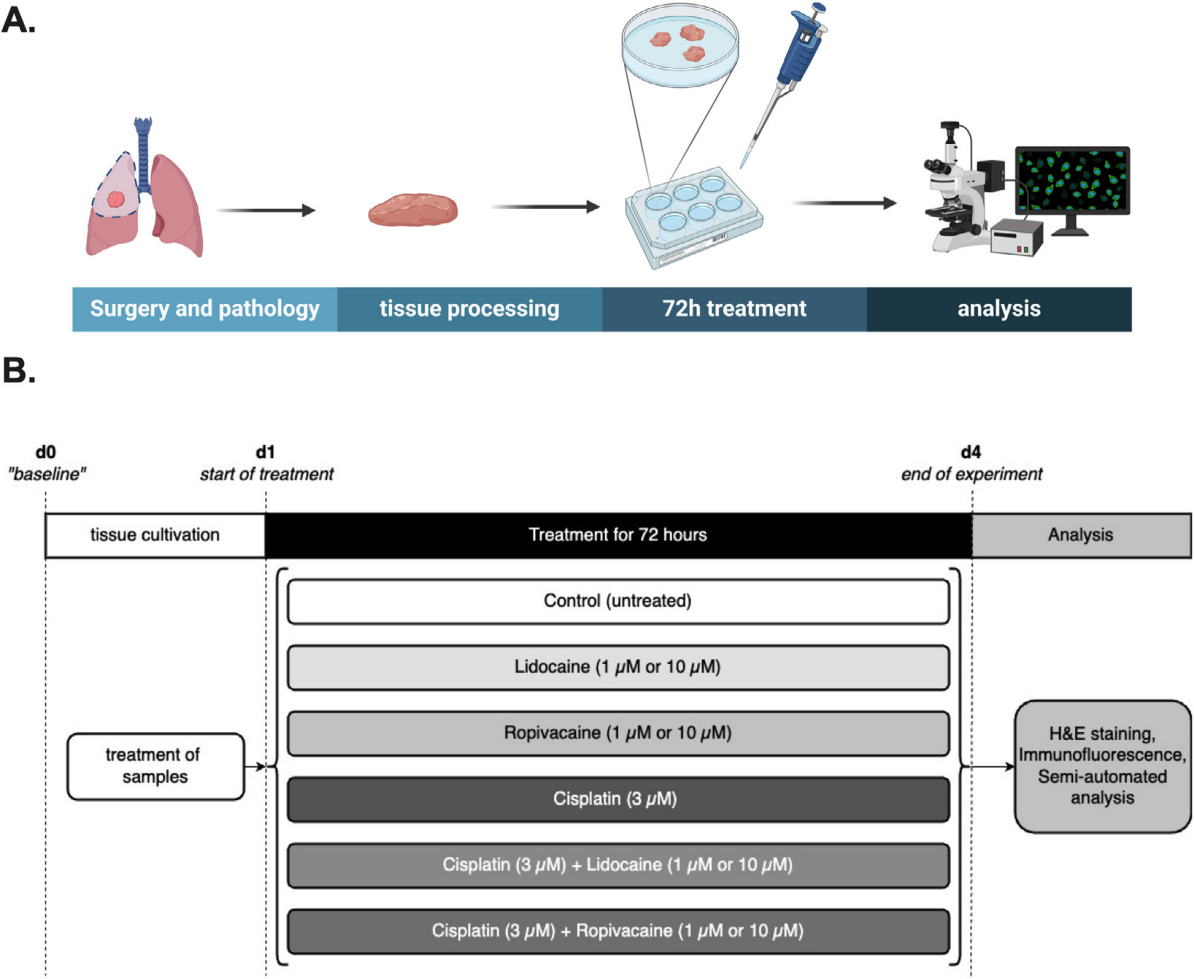
A) Schematic illustration of experimental workflow. B) Timeline of conducted experiments including the respective treatment of cultured tumor slices.

The tissue was immediately processed after surgical resection and pathological evaluation. Only NSCLaC were included and the previously described protocol was applied with some modifications.^[^ ^20^, ^22^ ^]^ In short, tumor samples were cut into slices of 350 µm using a tissue chopper (McIlwain TC752; Campden Instruments, Lafayette, IN, USA). Tissue slice diameter was then standardized by using a 3 mm punching tool (kai Europe, Solingen, Germany) and three tissue samples were chosen to generate tissue heterogeneity in all conditions. Tissue slices were cultivated in six‐well plates on membrane inserts (Millipore Corporation, Billerica, MA, USA) and were incubated under standardized conditions of 37 °C and 5% CO_2_. Tissue cultures were fixed overnight using 4% paraformaldehyde (16% Formaldehyde Solution, Thermo Fisher Scientific, Rockford, IL, USA) on the day of preparation (baseline, d0) and after treatment over 72 h (see section on experimental setup and treatment below). The workflow is visualized in **Figure** **1**A.

### 2.2 Experimental Setup and Treatment

Tissue slices were cultivated for 24 h in culture medium containing RMPI‐1640 (Gibco, Life Technologies, Paisly, UK), 10% fetal calf serum (Thermo Fisher Scientific, Waltham, MA, USA), 1% L‐Glutamine (Thermo Fisher Scientific, 200 mm) and 1% penicillin/streptomycin (Merck, Darmstadt, Germany; 10 000 U penicillin /10 mg mL^−1^ streptomycin in 0.9% NaCl) in order to adapt the tissue to culture conditions. Subsequently (at timepoint d1, Figure 1B), PDTC were treated for 72 hours with either lidocaine (diluted in normal saline 0.9%, final concentration 1 or 10 µm; mibe, Brehna, Germany) or ropivacaine (diluted in normal saline 0.9%, final concentration 1 or 10 µm; Sintetica, Münster, Germany) or cisplatin (3 µm, Cisplatin NeoCorp, Hexal AG, Holzkirchen, Germany) only or in combination with the chemotherapeutic agent. Previously, dose‐dependency as well as efficiency of cisplatin was tested in PDTCs of NSCLC and the concentration of 3 µm of cisplatin was found to be both efficient and close to clinical as well as to biologic reality.^[^ ^20^, ^23^ ^]^ The prolongated incubation time was chosen to reveal adaptations of the TME to treatment. Untreated slices served as control and culture media was exchanged every other day.

### 2.3 Staining Procedure

PFA‐fixated tissue slices were embedded in paraffin, cut into 6 µm sections and brought up on microscope slides. Hematoxylin and Eosin staining (H&E) was performed to assess morphology and tissue viability. Immunofluorescence stainings were conducted in order to analyze tumor cell count as well as proliferating and apoptotic cells. Furthermore, ICAM‐1 expression, CD68 and CD163 expression on TAM were evaluated in order to differentiate M1 and M2 macrophages.^[^ ^16^ ^]^ Briefly, after de‐paraffination and antigen retrieval, slides were washed with 0.3% PBS/TritonX and blocked with 5% normal goat serum (Jackson ImmunoResearch, Ely, Cambridgeshire, UK) for 30 min. Primary antibodys against Ki67 (1:200, rabbit, DCS, Hamburg, Germany), cleaved PARP (1:100, rabbit, Abcam, Cambridge, UK), cytokeratins AE1/AE3 (1:100, mouse, BioGenex, Fremont, CA, USA), ICAM‐1 (1:300, rabbit, Cell Signaling Technology, Danvers, MA, USA), CD68 (1:500, mouse, Dako) and CD163 (1:1000, rabbit, Abcam, Cambridge, UK) were diluted in 0.5% bovine serum albumine (Roth, Karlsruhe, Germany) and incubated at 4 °C overnight. Cell nuclei were stained with Hoechst 33342 (Sigma–Aldrich, St. Louis, MO, USA). Slides were rinsed with 0.3% PBS/TritonX and labeled with secondary antibodies Alexa Fluor goat‐anti‐rabbit 568 or 647, goat‐anti‐mouse 568 or 647, goat‐anti‐rat 647 for 1 h (Invitrogen, Carlsbad, CA, USA), respectively.

### 2.4 Analysis of Stained PDTC

H&E sections were analyzed using slide scans (Pannoramic SCAN and Pannoramic Viewer, 3D Histech, Budapest, Hungary). Up to five representative pictures per slice were taken from fluorescent sections with an Olympus BX51 fluorescent microscope (Magnification 40x, Olympus Deutschland, Hamburg, Germany). Immunofluorescence staining was evaluated by counting positive pixels via specific algorithms for each staining with the ImageJ software adapted to NSCLC tissue (modified from Sönnichsen and colleagues,^[^ ^22^ ^]^ Figure S1, Supporting Information). Total (Hoechst positive), proliferating (Hoechst and Ki67 positive), apoptotic (Hoechst and cPARP positive) and tumor cell count (Hoechst and AE1/3 positive) were assessed for each picture and manually reevaluated to ensure high accuracy. Additionally, colocalization of tumor cells and proliferating/apoptotic cells was evaluated and set in proportion to overall tumor cell count. Values of all conditions were normalized to the untreated control group (CTR, set as 1.0) to determine the effects of the applied treatments. The proportion of ICAM‐1 positive tumor cells was also counted using the colocalization function. The ratio of CD163 positive TAM in the overall macrophage population was calculated in proportion of CD163 (M2 phenotype) and CD68 (M1 phenotype) positive segmented pixels in the picture (Figure S2, Supporting Information). The number of apoptotic and proliferating tumor cells, respectively, in each condition was also normalized to the corresponding untreated tissue (CTR, set as 1.0).

### 2.5 Statistical Analysis

Values obtained from specific assessment were normalized to untreated cells (control) of the same experiment. Normal distribution was assessed using the Shapiro–Wilk test. Normally distributed data of ICAM‐1 expression and CD163/CD68 were analyzed further with two‐way ANOVA with *post‐hoc* testing using the two‐stage linear step‐up procedure of Benjamini, Krieger and Yekutieli or the Bonferroni method where appropriate in order to control the false discovery rate^[^ ^24^ ^]^ and with the LA and the absence or presence of cisplatin as factors to be tested. The F ratio with the degrees of freedom for each of these factors and for the interaction between them are reported where appropriate. The absolute, i.e., not normalized, proliferation and apoptosis values were analyzed with a paired two‐sided t‐test. Non‐normally distributed data were analyzed using non‐parametric testing methods (Wilcoxon matched pairs signed rank test or Kruskal‐Wallis test with Benjamini, Krieger, and Yekutieli *post‐hoc* testing). All analyses have been conducted using GraphPad Prism software for Mac, Version 10.2.2 (GraphPad Software, La Jolla, CA, USA). A *p*‐value of less than 0.05 was considered to be statistically significant.

For further descriptive and statistical analysis, specimens were divided into Responders and NON‐RResponders in terms of proliferation and apoptosis. For each specimen, proliferation and apoptosis were considered separately. A response is characterized by a reduction of the tumor proliferation fraction (TP) and an increase of the tumor apoptotic fraction (TA) by/to 50% and above, respectively. The threshold of 50% has been used in several other studies evaluating the activity of cancer therapeutics ex vivo and has therefore been applied to the current as well.^[^ ^25^ ^]^

### 2.6 Ethics Approval Statement

The study was approved by the Ethics Committee of the Medical Faculty, University of Leipzig (Protocol No. 370–1316122013).

### 2.7 Patient Consent Statement

Written informed consent was obtained from all patients involved in this study. All research was performed in accordance with the Declaration of Helsinki.

### 2.8 Prior Presentations

115th Annual Meeting of the Anatomical Society of Germany, September 2123, 2021. 39th Annual ESRA Congress, June 2225, 2022.

### 2.9 Clinical Trial Registration

Not applicable.

## 3 Results

### 3.1 Patient Characteristics

A total of nine patient‐derived tumors were assessed during the final analysis, three PDTC had to be excluded due to extensive necrosis already at baseline. Baseline characteristics of the patients and their disease are summarized in **Table** **1**.

**Table 1:** Patient characteristics: Patient‐specific characteristics including age, gender, TNM classification, grading, Union internationale contre le cancer (UICC) stage of the disease and mutation status. Specimens with "non mutation" ("‐") status did not carry a standard mutation.

Experiment No.	Age [years], Gender [m/f]	TNM	Grading	UICC stage	Mutation
1	74, m	pT2b pN2 cM1b	G3	IVA	–
2	55, m	pT3 pN1 M0	G3	IIIA	–
3	85, m	pT4 pN1 M0	G2	IIIA	–
4	65, f	pT1 pN1 M0	G2	IIB	KRAS +
5	66, f	pT1 pN0 M0	G1	IA2	KRAS +
6	62, m	pT1 pN0 M0	G2	IA2	–
7	68, m	pT4 pN0 M0	G3	IIIA	–
8	52, f	pT1 pN0 M0	G2	IA1	–
9	69, m	pT2 pN0 M1	G2	IVA	–

### 3.2 Effects of LA on Tumor Cell Proliferation and Apoptosis in PDTC of NSCLaC

**Figure 2:**
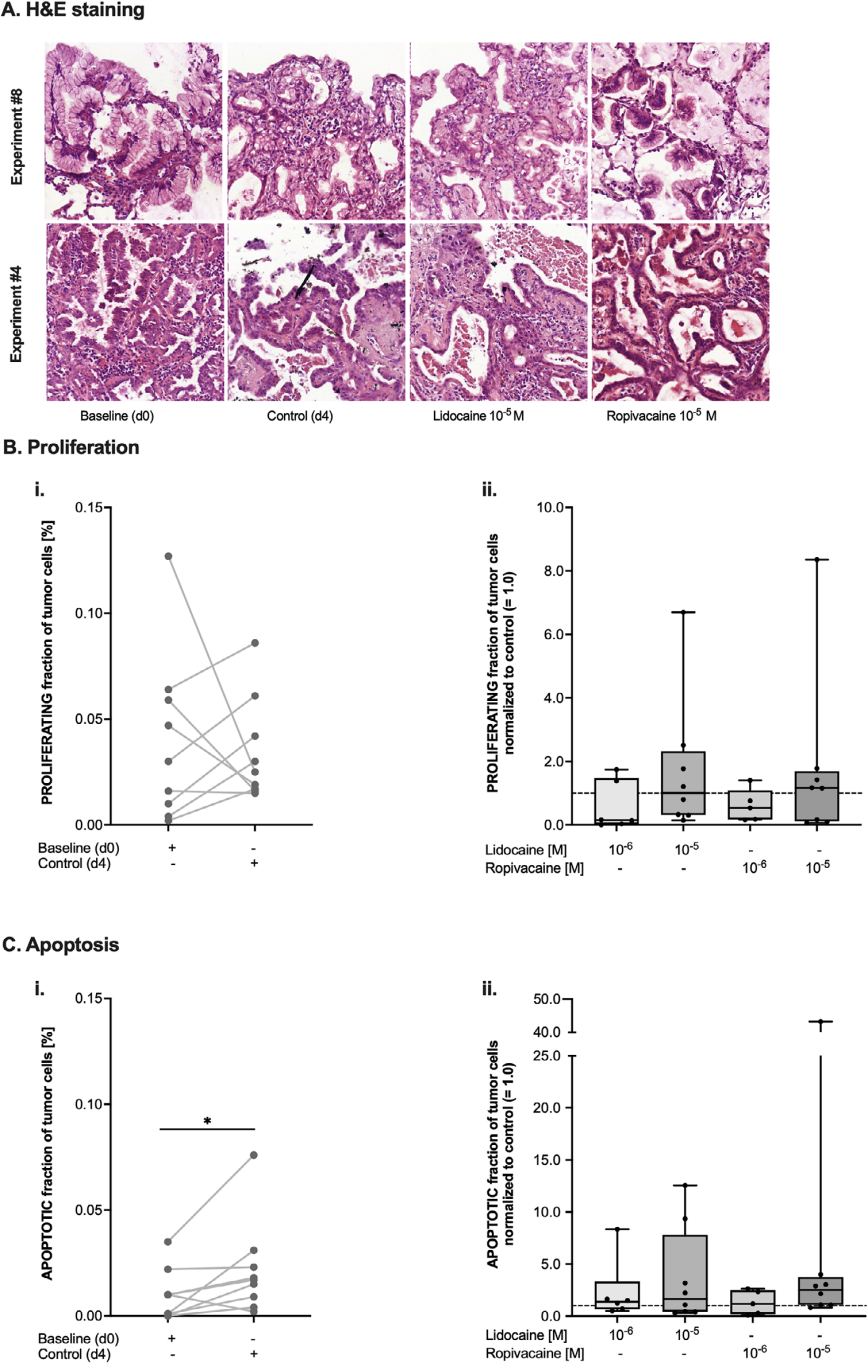
A) Representative display of cultured tumor slices from two different specimens at the beginning of the experiment (Baseline (d0)), after four days of culture but without any additional treatment (Control (d4)), or after four days of culture and 72 h of treatment with either lidocaine (Lidocaine 10^−5^ m) or ropivacaine (Ropivacaine 10^−5^ m) both at a final concentration of 10 µm. Staining with H&E. B) i) Absolute values of the fraction of proliferating tumor cells at the beginning of the experiment (Baseline (d0)) and after four days of culture (Control (d4)). Analysis with paired two‐sided t‐test. ii) Fraction of proliferating tumor cells after four days of culture normalized to control (= 1.0, dashed line) and after treatment with lidocaine or ropivacaine at different concentrations. Data are shown as boxplots with median and whiskers indicating minimum and maximum values. C) i) Absolute values of the fraction of apoptotic tumor cells at the beginning of the experiment (Baseline (d0)) and after four days of culture (Control (d4)) *p<0.05 as indicated (Wilcoxon matched pairs signed rank test). ii) Fraction of apoptotic tumor cells after four days of culture normalized to control (= 1.0, dashed line) and after treatment with lidocaine or ropivacaine at different concentrations. Data are shown as boxplots with median and whiskers indicating minimum and maximum values.

Lidocaine and ropivacaine were tested on PDTCs from surgical resections at final concentrations of 1 and 10 µm over 72 h. Representative images of H&E‐stained tumor slices are shown in **Figure** **2**A: All evaluated PDTCs (n = 9) maintained their original tissue morphology and architecture including the individual tumor ex vivo independent of the applied treatment. While there was a significant heterogeneity between proliferation rates at d0 and d4, the observed differences did not reach statistical significance (*p* = 0.731, Figure 2Bi). Treatment with the two different local anesthetics also did not alter proliferation rates when assessing all available experiments (*p* = 0.416, Figure 2Bii). The fraction of apoptotic tumor cells of individual tumors increased significantly from d0 to d4, while still being very low (median 0.01% at d0 vs 0.017% at d4, *p* = 0.04, Figure 2Ci). No statistically significant effect of local anesthetics on the rate of apoptosis could be demonstrated (*p* = 0.207, Figure 2Cii). There was a large range in the rates of both proliferation and apoptosis, especially after treatment with either lidocaine or ropivacaine (Figure 2Bii,Cii). A representative example of this heterogeneity is shown in Figure S3 (Supporting Information).

### 3.3 Categorization of Responding and Non‐Responding PDTC

Due to the observed extensive range in the response to LA treatment, the results were evaluated further and the different specimens were classified as Responders, when there was either a reduction of more than 50% in the proliferation rate or an increase in apoptosis of more than 50% compared to control after treatment with any of the local anesthetics.^[^ ^25^ ^]^ **Table** **2** shows the distribution of the experiments regarding their status as Responders or NON‐Responders.

**Table 2:** Classification of individual tumor specimens as responders or non‐responders regarding proliferation and apoptosis after treatment with local anesthetics. Experiments were classified as responders, when there was either a reduction of more than 50% in the proliferation rate or an increase in apoptosis of more than 50% compared to control after treatment with any of the local anesthetics. Experiment 1 was excluded, as there were no results regarding the treatment with local anesthetics alone.

Item	Category	Experiment No.
*Proliferation*	Responders	2 / 3 / 5 / 6 / 9
	NON‐Responders	4 / 7 / 8
*Apoptosis*	Responders	2 / 3 / 4 / 6 / 9
	NON‐Responders	5 / 7 / 8

**Figure 3:**
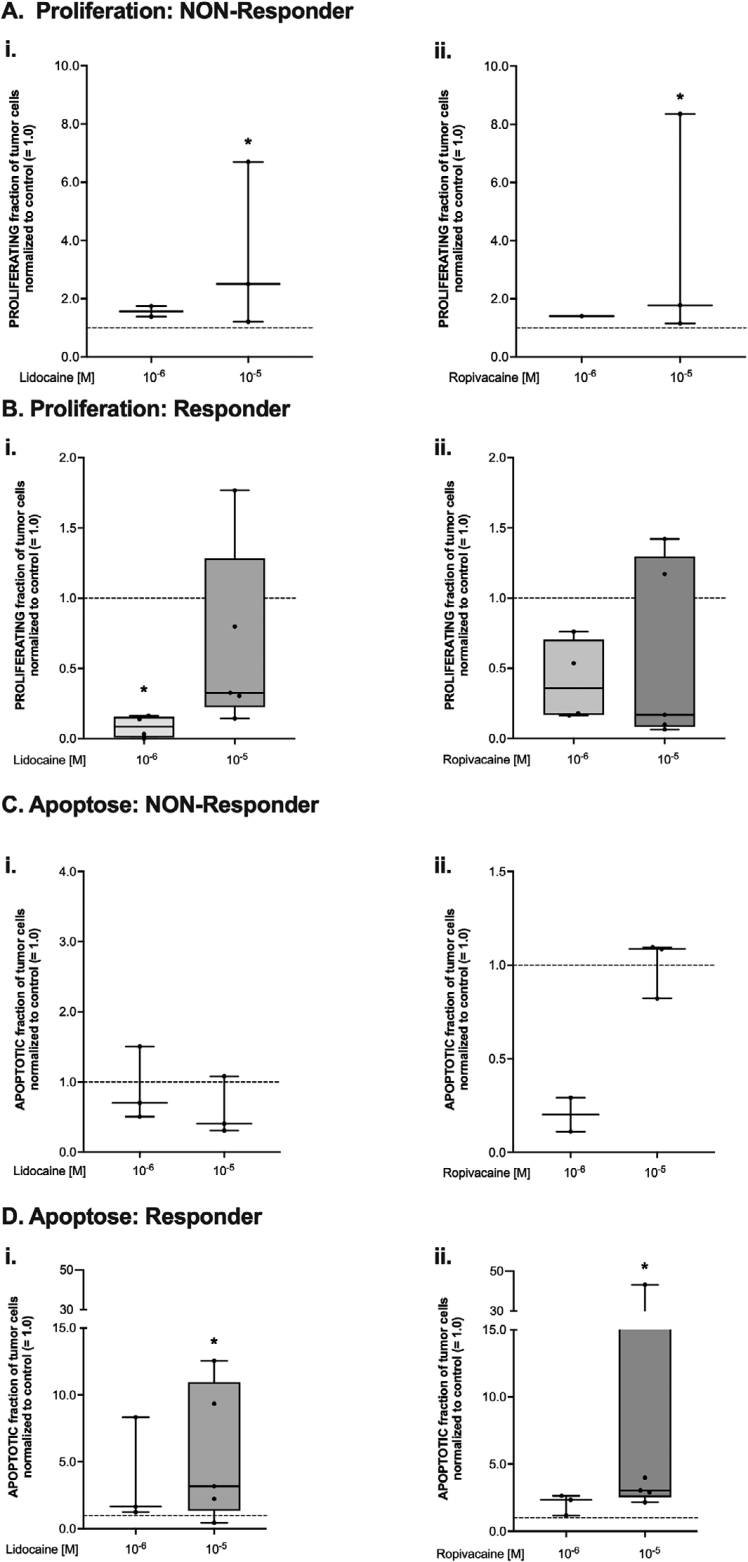
A) Fraction of proliferating tumor cells in *NON‐Responder* specimens after four days of culture normalized to control (= 1.0, dashed line) and after treatment with i) lidocaine or ii) ropivacaine at different concentrations. Data are shown as boxplots with median and whiskers indicating minimum and maximum values. *p<0.05 compared to control. B) Fraction of proliferating tumor cells in *Responder* specimens after four days of culture normalized to control (= 1.0, dashed line) and after treatment with i) lidocaine or ii) ropivacaine at different concentrations. Data are shown as boxplots with median and whiskers indicating minimum and maximum values. *p<0.05 compared to control. C) Fraction of apoptotic tumor cells in *NON‐Responder* specimens after four days of culture normalized to control (= 1.0, dashed line) and after treatment with i) lidocaine or ii) ropivacaine at different concentrations. Data are shown as boxplots with median and whiskers indicating minimum and maximum values. D) Fraction of apoptotic tumor cells in *Responder* specimens after four days of culture normalized to control (= 1.0, dashed line) and after treatment with i) lidocaine or ii) ropivacaine at different concentrations. Data are shown as boxplots with median and whiskers indicating minimum and maximum values. *p<0.05 compared to control. All statistical analyses were conducted using a Kruskal‐Wallis test with Benjamini, Krieger, and Yekutieli *post‐hoc* testing.

As clearly shown in **Figure** **3**A, lidocaine and ropivacaine led to a statistically significant increase in proliferation in the NON‐Responder tumors at the 10 µm concentration compared to control (median increase of 2.5‐fold for lidocaine, *p* = 0.026, Figure 3Ai and 1.8‐fold for ropivacaine, *p* = 0.031, Figure 3Aii). However, the same concentrations led to relevant decreases in the proliferation rates in responding specimens (67% for lidocaine 10 µm and 83% for ropivacaine 10 µm), although the observed difference did not reach statistical significance (*p* = 0.216 for lidocaine, Figure 3Bi, and *p* = 0.216 for ropivacaine, Figure 3Bii). The lower concentrations of the LA seemed to be even more effective as lidocaine at 1 µm diminished tumor proliferation in the Responder group compared to control by a median of 91% (*p* = 0.003, Figure 3Bi).

Similar results could be obtained from the apoptosis assessment of NON‐Responders and Responders: In the NON‐Responder group, lidocaine and ropivacaine did not alter apoptosis rates significantly (*p* = 0.618 for lidocaine, *p* = 0.093 for ropivacaine, Figure 3C), whereas the 10 µm concentration of both LAs increased the median fraction of apoptotic tumor cells in the responder group by 3.2‐ (lidocaine, p = 0.037, Figure 3Di) and threefold (ropivacaine, *p* = 0.002, Figure 3Dii), respectively.

**Figure 4:**
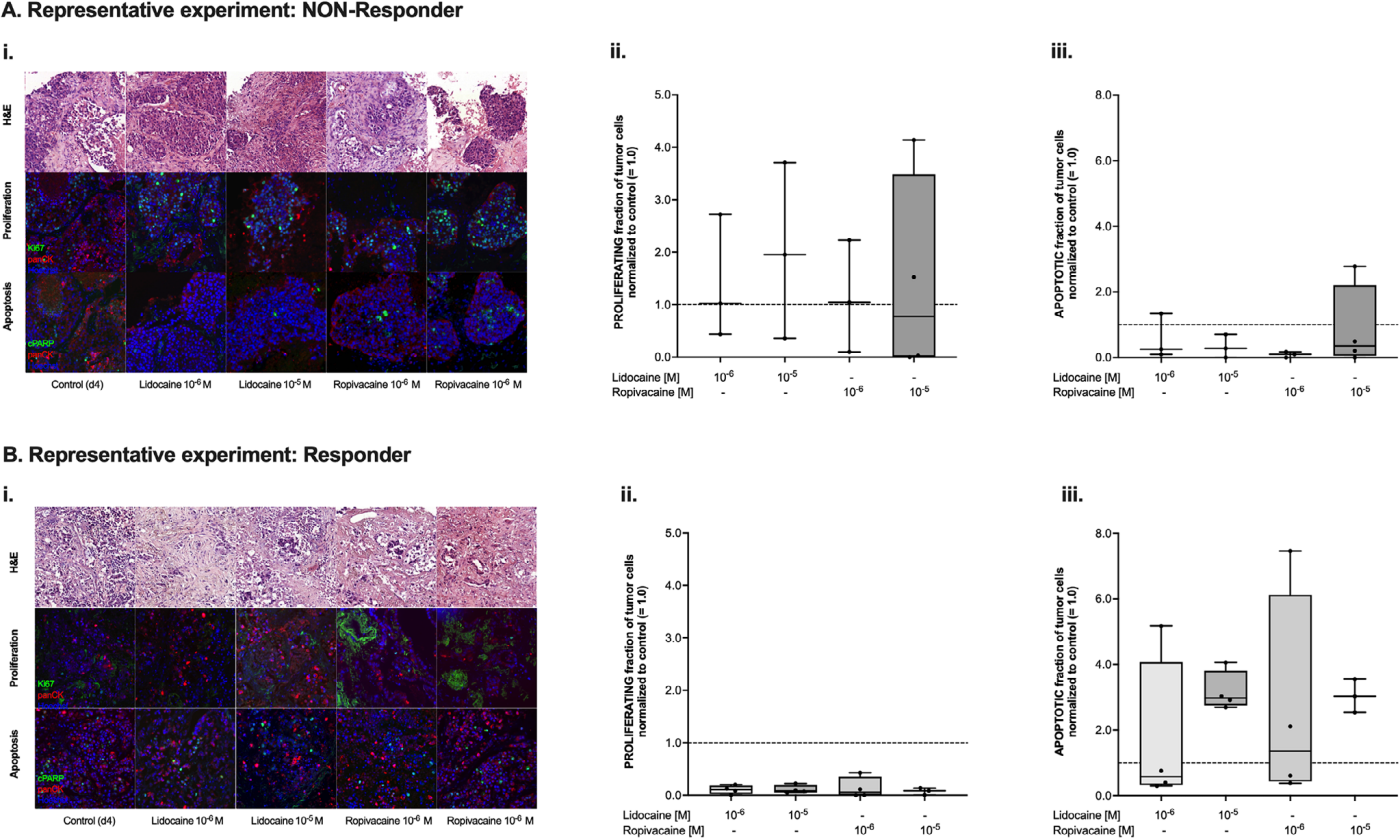
A) i) Representative images of slides from a *NON‐Responder* experiment stained after four days of culture but without any additional treatment (Control (d4)), or after four days of culture and 72 h of treatment with either lidocaine (Lidocaine 10^−6^ m, Lidocaine 10^−5^ m) or ropivacaine (Ropivacaine 10^−6^ m, Ropivacaine 10^−6^ m) both at a final concentration of 1 or 10 µm. Slides were either stained with H&E (first row), or with Ki67, panCK and Hoechst antibodies (second row) to assess proliferation or with cleaved PARP (cPARP), panCK and Hoechst antibodies (third row) for the quantification of apoptotic cells. ii) Fraction of proliferating tumor cells in this particular *NON‐Responder* specimen after four days of culture normalized to control (= 1.0, dashed line) and after treatment with lidocaine or ropivacaine at different concentrations. Data are shown as boxplots with median and whiskers indicating minimum and maximum values. iii) Fraction of apoptotic tumor cells in this particular *NON‐Responder* specimen after four days of culture normalized to control (= 1.0, dashed line) and after treatment with lidocaine or ropivacaine at different concentrations. Data are shown as boxplots with median and whiskers indicating minimum and maximum values. B) i) Representative images of slides from a *Responder* experiment stained after four days of culture but without any additional treatment (Control (d4)), or after four days of culture and 72 h of treatment with either lidocaine (Lidocaine 10^−6^ m, Lidocaine 10^−5^ m) or ropivacaine (Ropivacaine 10^−6^ m, Ropivacaine 10^−6^ m) both at a final concentration of 1 or 10 µm. Slides were either stained with H&E (first row), or with Ki67, panCK, and Hoechst antibodies (second row) to assess proliferation or with cleaved PARP (cPARP), panCK and Hoechst antibodies (third row) for the quantification of apoptotic cells. ii) Fraction of proliferating tumor cells in this particular *Responder* specimen after four days of culture normalized to control (= 1.0, dashed line) and after treatment with lidocaine or ropivacaine at different concentrations. Data are shown as boxplots with median and whiskers indicating minimum and maximum values. iii) Fraction of apoptotic tumor cells in this particular *Responder* specimen after four days of culture normalized to control (= 1.0, dashed line) and after treatment with lidocaine or ropivacaine at different concentrations. Data are shown as boxplots with median and whiskers indicating minimum and maximum values.

**Figure** **4** shows two representative experiments – one from the NON‐Responder (Figure 4A) and one from the Responder group (Figure 4B). Both tissue specimens had the same UICC classification (IIIA). In the NON‐Responder PDTC the density structure of the tumor remained high after treatment, preserving a physical barrier around the tumor cell nets that provides chemo‐resistance and reduces immune cell infiltration (experiment #7, Figure 4Ai). The specimen also showed a heterogeneous tumor proliferation rate in each tissue slice investigated, suggesting the differential regulation of tumor proliferation depending on the tumor microenvironment and tumor cell population (Figure 4Aii). Here, every datapoint represents one of the assessed 3–4 tissue slices (Figure 4Aii). Tumor apoptosis was evenly reduced in all investigated tissues after LA treatment as shown in Figure 4Aiii.

In contrast to the findings in the non‐responder PDTC, experiment #2 exerted a clearly visible response to LA treatment: The quantification of the IF stainings shown in Figure 4Bi demonstrated a significant decrease in tumor proliferation in all LA‐treated tissue slices (Figure 4Bii). The same tissue specimen also showed a dose‐dependent increase in tumor apoptosis (Figure 4Biii) after administration of either one of the LA.

### 3.4 PDTC Response of NSCLaC Specimen to Cisplatin Alone and in Combination with LA

**Figure 5:**
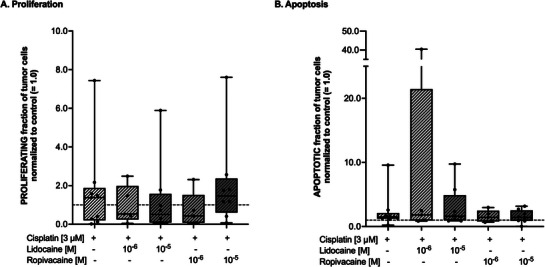
A) Fraction of proliferating tumor cells in cultured tumor slices after four days of culture normalized to control (= 1.0, dashed line) and after treatment with lidocaine or ropivacaine at different concentrations in combination with Cis‐Platin (final concentration 3 µm). Data are shown as boxplots with median and whiskers indicating minimum and maximum values. B) Fraction of apoptotic tumor cells in cultured tumor slices after four days of culture normalized to control (= 1.0, dashed line) and after treatment with lidocaine or ropivacaine at different concentrations in combination with Cis‐Platin (final concentration 3 µm). Data are shown as boxplots with median and whiskers indicating minimum and maximum values. All statistical analyses were conducted using a Kruskal–Wallis test with Benjamini, Krieger, and Yekutieli *post‐hoc* testing.

To investigate whether LA might exert a synergistic effect with a chemotherapeutic agent, PDTC specimens were incubated with cisplatin (final concentration 3 µm) alone or in combination with either one of the LA. Cisplatin increased the median proliferation rate (by 38%, **Figure** **5**A) over all experiments. There was, however, a large range in the values (minimum–maximum 0.018–7.435), so that this increase did not reach statistical significance (*p* = 0.912, Figure 5A). This observed scatter in the cisplatin response could also be noticed in the combined treatment of cisplatin together with the LA: Combined treatment with lidocaine led to a decrease in median proliferation of 47% (1 µm, *p* = 0.695) and 49% (10 µm, *p* = 0.315) without reaching statistical significance. Similar results could be obtained from the combined treatment of cisplatin together with ropivacaine at the 1 µm concentration (57% decrease, *p* = 0.287, all Figure 5A).

Compared to control, cisplatin monotherapy slightly induced tumor cell apoptosis, again with a large range in the observed values (median 49% increase, minimum–maximum 0.214–9.571, *p* = 0.053, Figure 5B). Combined treatment of LA and cisplatin also led to an increase in median apoptosis rates without reaching statistical significance and with no detectable difference compared to cisplatin alone (all Figure 5B). H&E stainings of cisplatin (3 µm) and low dose LA treated tumor specimen did only demonstrate minor alterations (Figure S3, Supporting Information).

### 3.5 ICAM‐1 Expression by Tumor Cells after LA Treatment in Absence or Presence of Cisplatin

**Figure 6:**
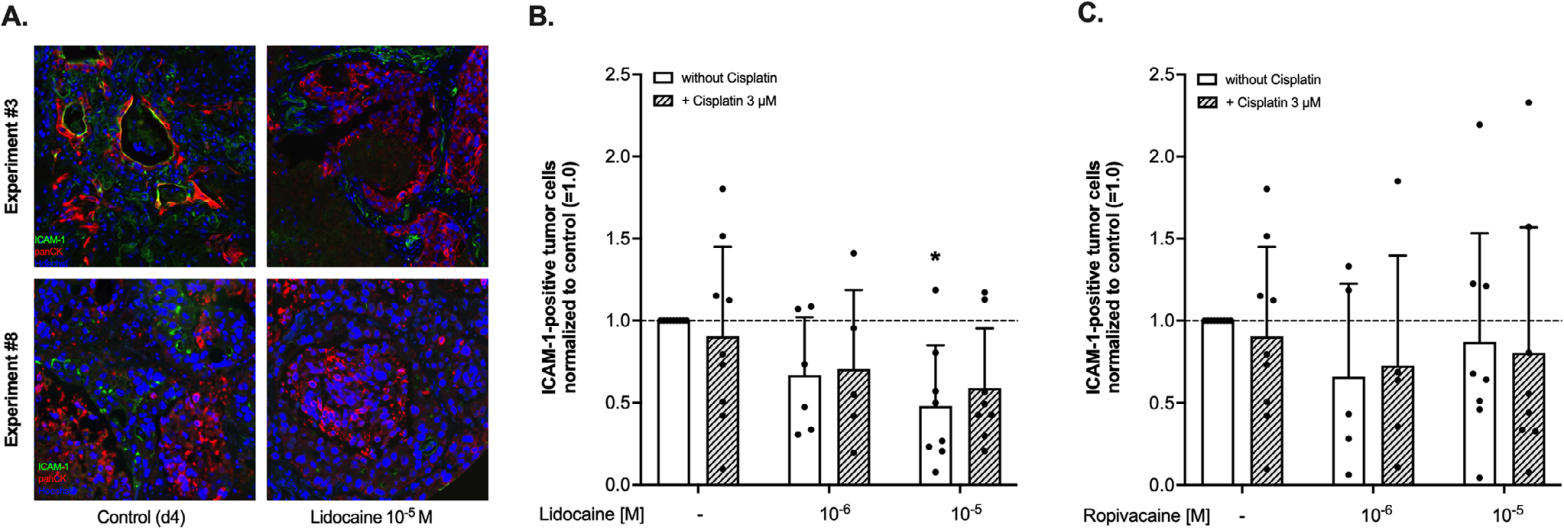
A) Representative display of cultured tumor slices from two different specimens after four days of culture but without any additional treatment (Control (d4)), or after four days of culture and 72 h of treatment with either lidocaine (Lidocaine 10^−5^ m) at a final concentration of 10 µm. Slides were stained with intercellular adhesion molecule‐1 (ICAM‐1), panCK, and Hoechst antibodies. B) Fraction of ICAM‐1‐positive tumor cells in cultured tumor slices after four days of culture normalized to control (= 1.0, dashed line) and after treatment with lidocaine at different concentrations in absence (white bars) or presence (striped bars) of Cis‐Platin (final concentration 3 µm). Data are shown as mean with corresponding standard deviation (SD). C) Fraction of ICAM‐1‐positive tumor cells in cultured tumor slices after four days of culture normalized to control (= 1.0, dashed line) and after treatment with ropivacaine at different concentrations in absence (white bars) or presence (striped bars) of Cis‐Platin (final concentration 3 µm). Data are shown as mean with corresponding SD. All statistical analyses were conducted utilizing a two‐way ANOVA with *post‐hoc* testing using the two‐stage linear step‐up procedure of Benjamini, Krieger, and Yekutieli.

ICAM‐1 expression on tumor cells was assessed using IF stainings as shown in **Figure** **6**A. Analysis with a two‐way ANOVA revealed a significant effect of lidocaine (F(2,39) = 5.095, *p* = 0.011), whereas the 10 µm concentration of the drug also significantly reduced ICAM‐1 expression compared to control (mean reduction 52%, p = 0.009). No significant effect of cisplatin on ICAM‐1 expression could be detected (F(1,39) = 0.02, *p* = 0.884. all Figure 6B).

Treatment with ropivacaine only also led to a decrease in ICAM‐1 expression, however, the difference did also not reach statistical significance (34% reduction by 1 µm, *p* = 0.295 and 13% reduction by 10 µm, *p* = 0.647,). Again the effect of cisplatin was negligible (9% reduction compared to control, F(1,38) = 0.03, *p* = 0.864) and showed a large heterogeneity between the different specimens (all Figure 6C).

### 3.6 CD163‐Positive Macrophages after LA Treatment in Absence or Presence of Cisplatin

**Figure 7:**
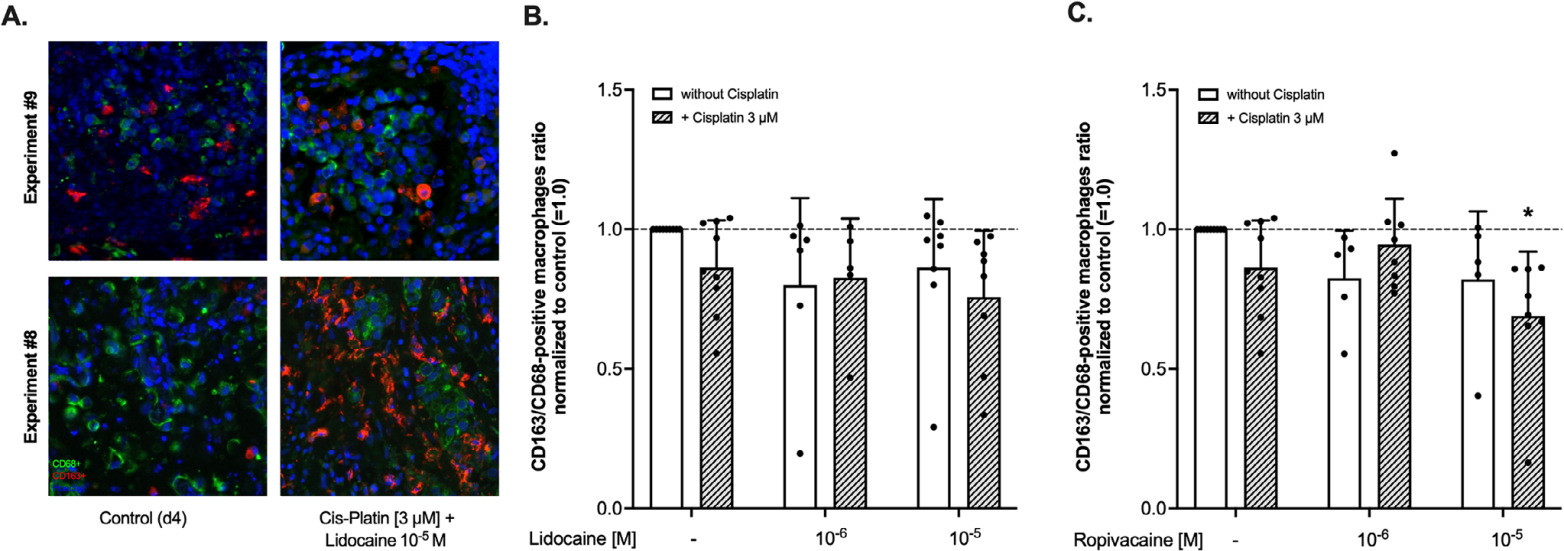
A) Representative display of cultured tumor slices from two different specimens after four days of culture but without any additional treatment (Control (d4)), or after four days of culture and 72 h of treatment with lidocaine and Cis‐Platin (Cis‐Platin [3 µm] + Lidocaine 10^−5^ m. Slides were stained with CD68, CD163, and Hoechst antibodies. B) Fraction of CD163‐ and CD68‐positive tumor cells in cultured tumor slices after four days of culture normalized to control (= 1.0, dashed line) and after treatment with lidocaine at different concentrations in absence (white bars) or presence (striped bars) of Cis‐Platin (final concentration 3 µm). Data are shown as mean with corresponding standard deviation (SD). C) Fraction of CD163‐ and CD68‐positive tumor cells in cultured tumor slices after four days of culture normalized to control (= 1.0, dashed line) and after treatment with ropivacaine at different concentrations in absence (white bars) or presence (striped bars) of Cis‐Platin (final concentration 3 µm). Data are shown as mean with corresponding SD. *p<0.05 compared to control. All statistical analyses were conducted utilizing a two‐way ANOVA with *post‐hoc* testing using the two‐stage linear step‐up procedure of Benjamini, Krieger, and Yekutieli.

Applying the simplified approach of dividing the macrophage population into an anti‐tumoral, pro‐inflammatory M1 and pro‐tumoral M2 phenotype, the fraction of CD163‐positive M2 macrophage in relation to the total macrophage population was assessed. Values above 1.0 indicate the predominance of M2, generating a pro‐tumoral and immunosuppressive micromilieu.^[^ ^26^ ^]^ Representative IF images are demonstrated in **Figure** **7**A. Analysis using two‐way ANOVA revealed no significant effect of neither lidocaine (F(2,39) = 1.781, *p* = 0.182) nor cisplatin (F(1,39) = 1.2, *p* = 0.267, both Figure 7B), although lidocaine alone reduced the fraction of CD163‐positive macrophages by 20% (1 µm) and 14% (10 µm). In contrast, ropivacaine was shown to have a significant impact in the two‐way ANOVA (F(2,38) = 3.8, *p* = 0.03, Figure 7C). *Post‐hoc* testing also revealed a significant decrease compared to control as induced by ropivacaine at 10 µm in combination with cisplatin (*p*<0.001, Figure 7C). A synergistic effect of cisplatin together with either one of the LA could not be detected, i.e., the interaction of the terms was not significant in the two‐way ANOVA (lidocaine:cisplatin F(2,39) = 0.5, *p* = 0.584 and ropivacaine:cisplatin F(2,38) = 2.3, *p* = 0.106).

### 3.7 ICAM‐1 Expression and CD163‐Positive Macrophages of Responding and Non‐Responding PDTC

**Figure 8:**
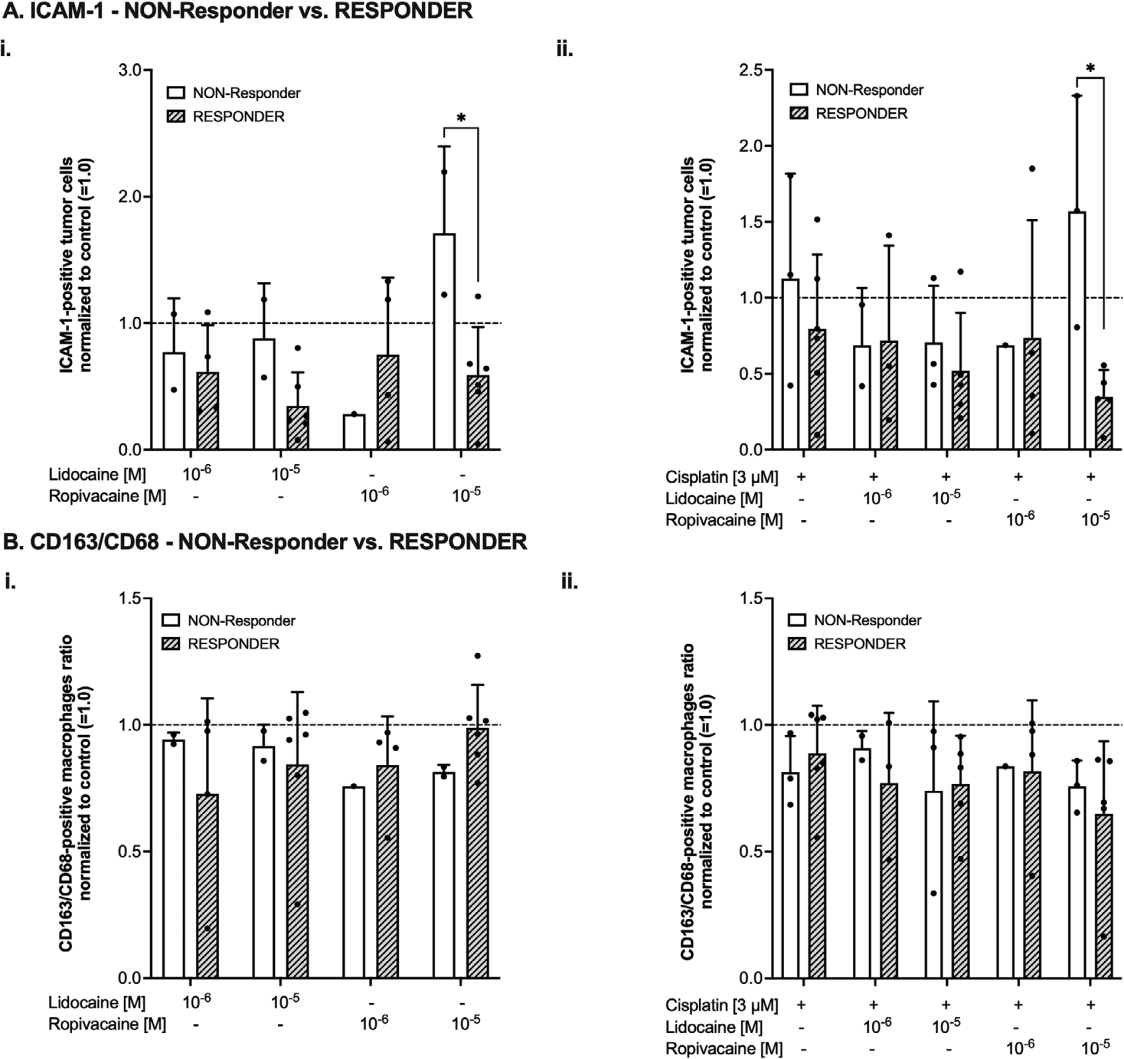
A) i) Fraction of ICAM‐1‐positive tumor cells in cultured tumor slices after four days of culture normalized to control (= 1.0, dashed line) and after treatment with lidocaine or ropivacaine at different concentrations in NON‐Responder (white bars) or Responder specimens (striped bars). Data are shown as mean with corresponding standard deviation (SD). *p < 0.05 as indicated. ii) Fraction of ICAM‐1‐positive tumor cells in cultured tumor slices after four days of culture normalized to control (= 1.0, dashed line) and after treatment with lidocaine or ropivacaine at different concentrations in combination with Cis‐Platin (final concentration 3 µm) in NON‐Responder (white bars) or Responder specimens (striped bars). Data are shown as mean with corresponding SD. *p<0.05 as indicated. B) i) Fraction of CD163‐ and CD68‐positive tumor cells in cultured tumor slices after four days of culture normalized to control (= 1.0, dashed line) and after treatment with lidocaine or ropivacaine at different concentrations in NON‐Responder (white bars) or Responder specimens (striped bars). Data are shown as mean with corresponding standard deviation (SD). ii) Fraction of CD163‐ and CD68‐positive tumor cells in cultured tumor slices after four days of culture normalized to control (= 1.0, dashed line) and after treatment with lidocaine or ropivacaine at different concentrations in combination with Cis‐Platin (final concentration 3 µm) in NON‐Responder (white bars) or Responder specimens (striped bars). Data are shown as mean with corresponding SD. All statistical analyses were conducted utilizing a two‐way ANOVA with Bonferroni *post‐hoc* testing.

Based on the previously determined responding and non‐responding specimens (see Table 2), correlations between responder status and ICAM‐1 expression or macrophage polarization were also investigated. In Responder specimens, the number of ICAM‐1‐positive tumor cells decreased dose‐dependently with both lidocaine and ropivacaine (mean ± SD for lidocaine 1 µm: 62 ± 37% of control, 10 µm: 35 ± 26%; ropivacaine 1 µm: 75 ± 61%, 10 µm: 59 ± 38%, **Figure** **8**Ai). A two‐ANOVA demonstrated that neither the LA treatment (F(3,19) = 2.3, *p* = 0.108, nor the responder status (F(1,19) = 2.9, *p* = 0.101) or the interaction of the terms (F(3,19) = 2.7, *p* = 0.072) reached statistical significance. However, the post‐hoc analysis revealed a statistically significant effect of ropivacaine at 10 µm, when comparing Responders to NON‐Responders (Responders 59 ± 38% of control vs NON‐RResponders 117 ± 69% of control, *p* = 0.018, all Figure 8Ai). Similar results could be obtained from the analysis of probes treated with cisplatin ± LA (Figure 8Aii): There was again a decrease of ICAM‐1 expression in most of the different experiments, which reached statistical significance in the comparison of esponders and NON‐RResponders after treatment of the slices with cisplatin plus ropivacaine at a final concentration of 10 µm (*p* = 0.021, Figure 8Aii).

Distinct differences between Responders and NON‐Responders were also observed in macrophage polarization. In Responders, administration of LA only led to a shift toward the CD68‐positive phenotype, i.e., less CD163‐postive macrophages could be detected (Figure 8Bi). However, the observed alterations were all not statistically significant in terms of the treatment (F(3,19) = 0.1, *p* = 0.921), the responder status (F(1,19) = 0, *p* = 0.945) or the interaction of the terms (F(3,19) = 0.6, *p* = 0.568, all Figure 8Bi). A shift toward the M1 phenotype (less CD163‐positive macrophages) was detected in both NON‐Responders and Responders after treatment with cisplatin ± LA (Figure 8Bii). However, in contrast to LA monotherapy, combined treatment of cisplatin and the LA revealed a dose‐dependent shift toward M1 in the Responder group, which was even more pronounced at the 10 µm concentration of ropivacaine (reduction of 35% compared to control).

## 4 Discussion

By utilizing PDTCs of NSCLaC, the current exploratory study provides translational data regarding the beneficial effects of amide LA, lidocaine and ropivacaine, on tumor biology. The effect sizes observed after treatment of the cultured tumor slices with LA alone are comparable to those observed with the chemotherapeutic agent cisplatin, which is still an integral part of most adjuvant regimens.^[^ ^27^ ^]^

It could already be demonstrated in vitro that LA might bear potentially anti‐tumor and anti‐metastatic properties^[^ ^13^, ^28^, ^29^ ^]^ and that the observed effects might be due to the well‐established anti‐inflammatory effects of these substances.^[^ ^6^, ^14^, ^30^, ^31^ ^]^ Nevertheless, several retrospective analyses have suggested a potential role of LA in the prevention of tumor recurrence after tumor surgery,^[^ ^32^ ^]^ but there have also been reports showing no effect, including several randomized controlled trials.^[^ ^18^, ^33^ ^]^ The reason for this discrepancy is discussed elsewhere in detail, highlighting genetic tumor heterogeneity and the TME itself.^[^ ^34^ ^]^ To our knowledge, this is the first ex vivo study exploring effects of LA on PDTCs of NSCLC samples. The prolonged incubation period of 72 h was chosen to mimic an extended perioperative use of local anesthetics (either as a continuous epidural administration of ropivacaine or a prolonged intravenous infusion of lidocaine) with relevant systemic plasma concentrations of the drugs.^[^ ^35^, ^36^ ^]^

As PDTCs provide a very individual TME and a high inter‐and intra‐tumoral heterogeneity in comparison to other models, the response to treatment with LA was also heterogenous and did not show beneficial effects when analyzing the overall response of all assessed specimen as expected. These observations are also in accordance with the results of most of the clinical studies and the heterogeneity of the TME in the individual patient might therefore also be one of the reasons for the negative results.^[^ ^18^, ^33^ ^]^ However, independent and individual evaluation of each PDTC showed that LA treatment led to a favorable alteration (of more than 50%^[^ ^25^ ^]^) of tumor proliferation and tumor apoptosis compared to their untreated PDTCs in more than 50% of the investigated patient samples. This effect was even more pronounced with the clinical relevant concentration of 1 µm. As the LA was also administered only into culture media and PDTCs are placed on a membrane on top of the media, LA tissue concentration is supposed to be even lower.

Separating PDTCs toward their response to LA treatment (Responder vs Non‐Responder), we also investigated ICAM‐1 expression and alterations in TAM population and polarization. ICAM‐1 expression does not only serve as a prognostic factor for outcome after treatment in cancer^[^ ^37^ ^]^ but also plays an important role in the mechanism by which LA might exert their anti‐metastatic properties.^[^ ^13^, ^28^ ^]^ In the analysis of all experiments, only high doses of lidocaine were able to significantly decrease ICAM‐1 expression, whereas ropivacaine and lidocaine at low concentrations did not reach statistical significance. ICAM‐1 expression in conditions supplemented with cisplatin displayed a high variance, but interestingly did not have any impact on ICAM‐1 expression regulation as provoked by LA. This was also independent of the fact whether a PDTC responded to cisplatin treatment or not. Previously published data, however, showed an effect of LA on ICAM‐1 expression either at higher concentrations of single LA administration or after a combination of the LA with an inflammatory stimulus, such as bacterial lipopolysaccharide (LPS) or tumor necrosis factor‐α (TNF‐α) in vitro.^[^ ^14^, ^30^ ^]^ Although the observed reductions as induced by lidocaine and ropivacaine did not reach statistical significance in the current study, mean ICAM‐1 expression demonstrated a trend toward a reduction compared to untreated controls. Knowing that ICAM‐1 is supposed to be a key element in mechanisms allowing tumors to escape immunosurveillance,^[^ ^37^ ^]^ the observed reduction in its expression by the amide‐LA suggests a potential mechanism, by which the drugs might exert anti‐metastatic potential.^[^ ^28^ ^]^ Further research is required to clearly identify signaling pathways and regulatory mechanisms underlying ICAM‐1 and the role of macrophage phenotype in tumor and metastasis progression.

Macrophage polarization upon LA treatment was further assessed as well, as macrophages not only exhibit different entry mechanisms for Ca^2+^, limiting their inflammatory potencies^[^ ^38^ ^]^ but can also shift toward a TAM polarization with anti‐tumoral properties, a mechanism that also involves ICAM‐1.^[^ ^39^ ^]^ No significant effects regarding a shift in TAM population were observed: Neither lidocaine nor ropivacaine induced a one‐directional shift of the number of CD68‐ and CD163‐positive cells. But differential analysis of Responder PDTCs to Non‐Responders revealed that amide‐LA treatment led to a more stable fraction of inflammatory macrophages. Using an inflammatory cellular model, it was previously demonstrated that another LA, neosaxitoxin, could effectively reduce LPS‐induced inflammation by sodium channel blockade in macrophages.^[^ ^40^, ^41^ ^]^ However, in the present system model we are not able to differentiate the direct effect of LA on macrophages itself. It is suggested that depending on the TAM polarization amide‐LA might cause differential effects, like the observed effects in ICAM‐1 expression. Contradicting literature exists whether ICAM‐1 promotes beneficial or negative macrophage polarization as several processes, such as phagocytosis, inflammation and wound healing might be involved.^[^ ^16^, ^42^, ^43^, ^44^ ^]^ However, Responder PDTCs demonstrated lower ICAM‐1 expression and mostly stable macrophage populations after treatment with the LA. Previous reports have demonstrated that LA might bear the ability to show a synergistic effect with chemotherapeutics, as LA were able to enhance the sensibility of, e.g., liver or colon cancer cells against cisplatin in vitro or in animal xenografts.^[^ ^45^, ^46^, ^47^ ^]^ However, in the current study no synergistic effects are observed. A larger cohort and potential future experiments with autologous leukocytes will allow to investigate specific actions of LA toward the interplay of ICAM‐1 expression, TAM polarization and tumor survival. Overall, the exact phenotype of the tumor, which might be susceptible to effective LA treatment, remains unknown. The evaluation of certain biomarkers and/or expression patterns might therefore be a promising approach for future investigations.

Moreover, in several of the assessed tumors, cisplatin showed very little to no effects on tumor proliferation or apoptosis, although the concentration used represents a realistic bioavailable concentration for deeper tissue regions of the clinical setting in combinational approaches, as tissue penetration and plasma concentrations are major hallmarks.^[^ ^20^, ^23^, ^48^ ^]^ The observed response rate in PDTCs, is also in accordance with the observation of an increase in chemoresistance, especially in patients with NSCLC.^[^ ^49^ ^]^ The underlying mechanism is still not completely understood and comprises a rather complex interaction of multiple factors and signaling pathways, including several characteristic gene mutations, such as the “gain of function mutation” of TP53, the p53 protein determining gene, a key regulator of apoptosis.^[^ ^49^ ^]^

During regular molecular pathology workup of tumors, the specimen were checked for mutations.^[^ ^50^ ^]^ Only two out of nine patients demonstrated KRAS (Kirsten rat sarcoma viral oncogene homolog) mutations. Interestingly, only these two cases, showed a diverse regulation of tumor proliferation and tumor apoptosis to amide‐LA treatment. KRAS is a frequent mutation in NSCLC and is thought to be an “oncogenic driver”, partially explaining the controverse tumor cell reactivity, as LA can inhibit phosphatidylinositol 3‐kinase (PI3K) activated by a KRAS mutation.^[^ ^13^, ^51^ ^]^

## 5 Conclusion 

The current study clearly shows the tremendous potential of amide LA regarding their anti‐tumor properties, despite the low number of patient samples included in this exploratory study. However, it is obvious that not all patients benefit from LA treatment and biomarkers for patient stratification are needed to be assessed in a bigger cohort. ICAM‐1 expression and macrophage polarization might play a role in the observed effects but the addition of autologous leukocytes in PDTCs should further determine the relevance of the presented findings. Future studies will therefore focus on the evaluation of the phenotype of Responders. By using this knowledge together with all the tumor‐related data provided by oncologists, one might hopefully become able to predict, which patients might benefit from perioperative treatment with LA and impact patient survival by utilizing a more personalized anesthesiologic approach for patients undergoing tumor surgery.

## Conflict of Interest

Tobias Piegeler received speakers honoraria from Edwards LifeSciences and CSL Behring unrelated to the current study.

## Supporting information



Supporting Information

## Data Availability

The data that support the findings of this study are available on request from the corresponding author. The data are not publicly available due to privacy or ethical restrictions.
