# Supplementary material for: Amide‐Linked Local Anesthetics Alter Tumor Biology in a Complex Human Tissue Model of Non‐Small Cell Lung Adenocarcinoma
Source: Adv Biol (Weinh). 2025 Sep 30;9(12):e00280. doi: 10.1002/adbi.202500280 (PMC12712772; doi:10.1002/adbi.202500280)
Supplement: Supplementary file 1 — Supporting Information [file ADBI-9-e00280-s001.pdf]

Supplemental Figure 1: Workflow background substraction

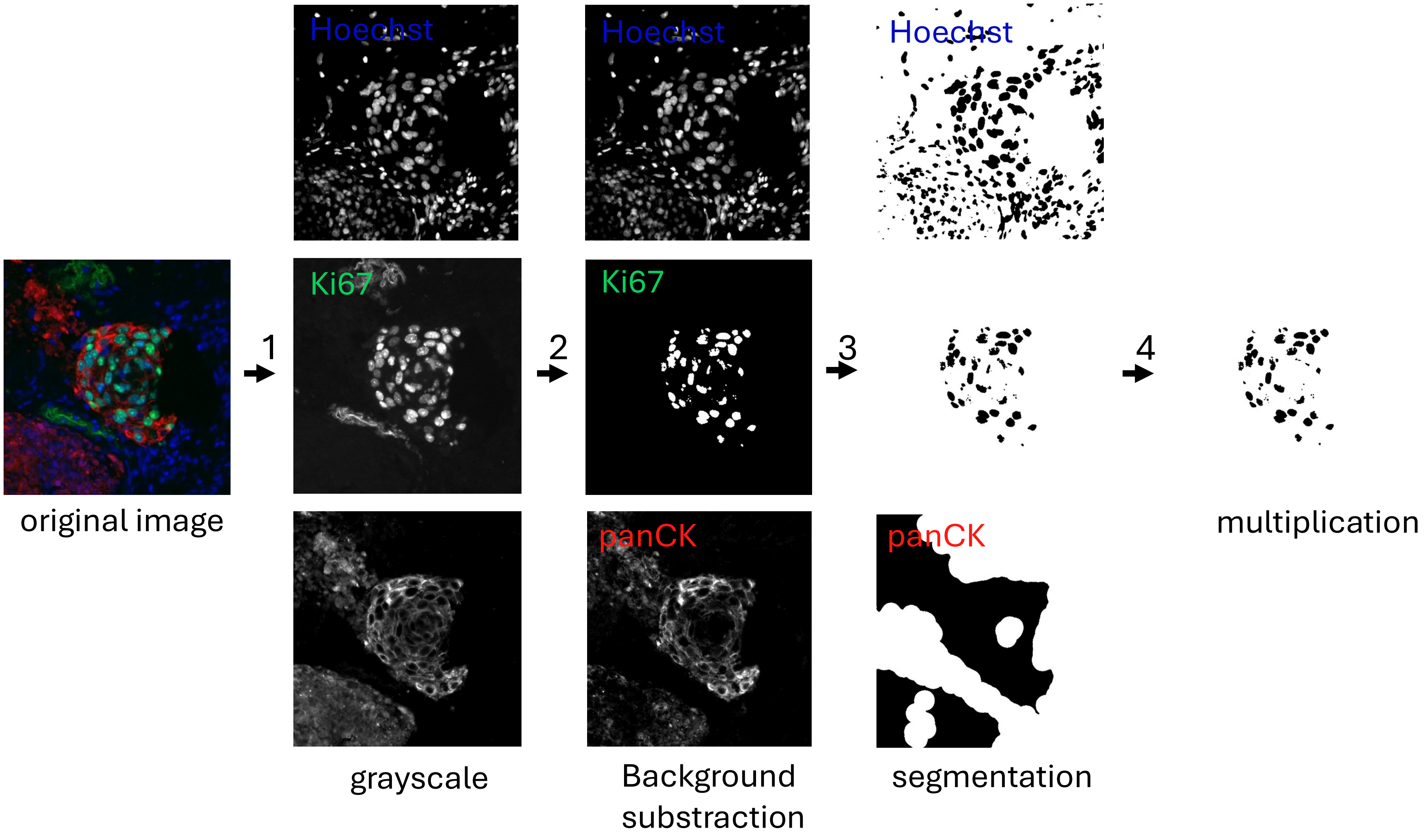

Supplemental Figure 1: Workflow for immunofluorescence analysis by ImageJ
Immunofluorescence images were analyzed by a staining specific algorithm in ImageJ adapted from Sönnichsen and colleagues (Clin Colorectal Cancer. 2018 Jun;17(2):e189-e199. doi: 10.1016/j.clcc.2017.11.002), adapted to lung tissue. Grayscale images were generated (1), background visible in the original image was substracted (2) and segmentation of positive pixel was performed (3). Total amount of proliferating or apoptotic tumor cells was calculated by multiplication. Macrophage population was calculated using the same algorithm in ImageJ, adapted to morphological features of macrophage phenotype.

Supplemental Figure 2: Macrophage ratio

i.

$$\text{M2 ratio: } \frac{CD163}{CD136 + CCD68}$$

ii.

Experiment	M2 ratio baseline (d0) in %	M2 ratio control (d4) in %
#1	37,7559839	35,4277137
#2	94,7938819	82,5232221
#3	93,990222	91,008725
#4	85,2833388	96,2520163
#5	96,4021785	92,2762299
#6	89,5810437	96,6497039
#7	50,9000822	87,5958386
#8	81,8573911	91,6446131
#9	27,4406493	44,0620948

Supplemental Figure 2: Total macrophage count and macrophage ratio
(i.) M2 ratio was calculated by dividing CD163+ count through total macrophage population.
(ii.) The M2 ratio was evaluated in baseline (d0) and control (d4) conditions. Intra-experimental analysis revealed elevations in the M2 ratio in some cases, primarily attributed to adaptive mechanisms. However, a stability of M2 ratio from d0 to d4 throughout the majority of experiments could be demonstrated.

Supplemental Figure 3: Tissue heterogeneity after local anesthetic treatment

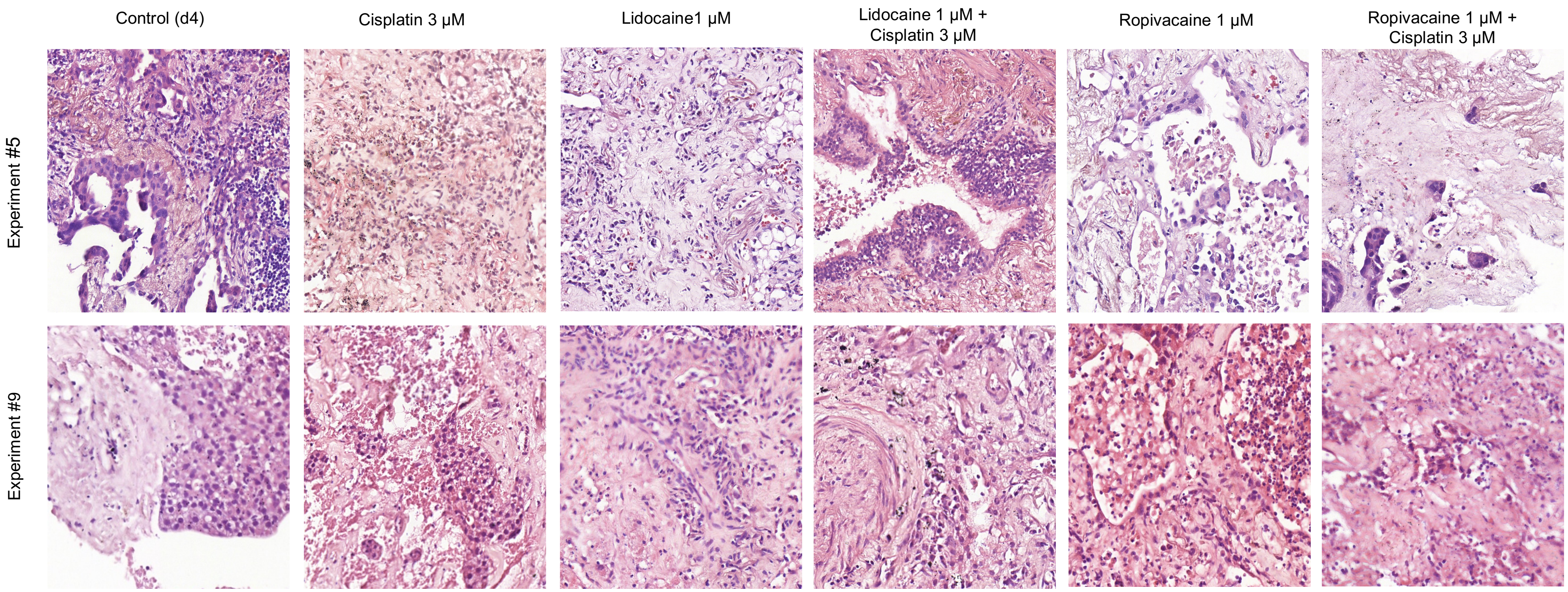

Supplemental Figure 3: Tissue heterogeneity after local anesthetic treatment
H&E stainings of two NSCLC specimen (experiment #5 and #9) demonstrate individual response to cisplatin and low dose local anesthetic treatment. Within individual experiments, different histomorphological responses regarding tumor cell vitality and tissue architecture were visible.
